# A Community Health Worker “logic model”: towards a theory of enhanced performance in low- and middle-income countries

**DOI:** 10.1186/1478-4491-12-56

**Published:** 2014-10-02

**Authors:** Joseph F Naimoli, Diana E Frymus, Tana Wuliji, Lynne M Franco, Martha H Newsome

**Affiliations:** United States Agency for International Development, 1300 Pennsylvania Avenue NW, Washington, DC USA; University Research Co., LLC, 7200 Wisconsin Avenue, Bethesda, MD USA; EnCompass LLC, 11426 Rockville Pike, Rockville, MD USA; World Vision International, 300 I St. NE, Washington, DC USA

**Keywords:** Causal thinking, Community health workers, Logic model, Performance, Policy, Programming

## Abstract

**Background:**

There has been a resurgence of interest in national Community Health Worker (CHW) programs in low- and middle-income countries (LMICs). A lack of strong research evidence persists, however, about the most efficient and effective strategies to ensure optimal, sustained performance of CHWs at scale. To facilitate learning and research to address this knowledge gap, the authors developed a generic CHW logic model that proposes a theoretical causal pathway to improved performance. The logic model draws upon available research and expert knowledge on CHWs in LMICs.

**Methods:**

Construction of the model entailed a multi-stage, inductive, two-year process. It began with the planning and implementation of a structured review of the existing research on community and health system support for enhanced CHW performance. It continued with a facilitated discussion of review findings with experts during a two-day consultation. The process culminated with the authors’ review of consultation-generated documentation, additional analysis, and production of multiple iterations of the model.

**Results:**

The generic CHW logic model posits that optimal CHW performance is a function of high quality CHW programming, which is reinforced, sustained, and brought to scale by robust, high-performing health and community systems, both of which mobilize inputs and put in place processes needed to fully achieve performance objectives. Multiple contextual factors can influence CHW programming, system functioning, and CHW performance.

**Conclusions:**

The model is a novel contribution to current thinking about CHWs. It places CHW performance at the center of the discussion about CHW programming, recognizes the strengths and limitations of discrete, targeted programs, and is comprehensive, reflecting the current state of both scientific and tacit knowledge about support for improving CHW performance. The model is also a practical tool that offers guidance for continuous learning about what works. Despite the model’s limitations and several challenges in translating the potential for learning into tangible learning, the CHW generic logic model provides a solid basis for exploring and testing a causal pathway to improved performance.

## Background

### Community Health Workers in low- and middle-income countries

The positive impact that Community Health Workers (CHWs) can have on people’s health and well-being in low- and middle-income countries (LMICs) is well-documented [1–4]. The final push toward achieving the Millennium Development Goals by 2015, current post-2015 discussions, and the introduction of Universal Health Coverage [5] have prompted many LMICs to increasingly invest in CHW programming in the hope of creating more accessible, equitable, and people-centered health systems [6]. A core challenge of CHW programming is how to ensure sustained, optimal performance at scale of this important cadre of the health workforce.

Available research evidence on the most efficient and effective strategies to ensure such performance, however, is weak [7]. Nevertheless, CHW programs continue to grow and expand, usually incorporating a variety of strategies to support performance with promising but uncertain effectiveness [8]. Consequently, more research is needed on how best to ensure optimal CHW performance at scale, particularly when LMIC governments are increasing domestic expenditures on health and donor support to the health sector is in a state of transition [9].

Although collective understanding of the definitive causal pathway to improved performance is limited, policy makers, program managers, practitioners, and the academic community can work together to develop theories about how to improve and sustain performance. The paper proposes such a theory, in the form of a generic CHW “logic model,” drawing upon available research and expert knowledge of CHWs in LMICs. Combining a somewhat patchwork collection of evidence (from studies in the published and gray literature) with the tacit knowledge of experts [10], and translating this knowledge into a practical tool for decision makers, is not unique to CHW performance, but is rather a recurrent challenge in on-going efforts to address the knowledge-practice gap in global public health [11–13].

### Logic models

Policy makers, program planners, project managers, and other analysts use logic models to communicate succinctly and visually the underlying theory of their policies and programs. Funnell and Rogers define a program theory as, “*an explicit theory or model of how an intervention, such as a project, a program, a strategy, an initiative, or a policy contributes to a chain of intermediate results and finally to the intended or observed outcomes*” [14]. A logic model maps the intended relationships and causal connections between what a program plans to do and what it hopes to achieve [15, 16]. A logic model commonly includes contextual factors that may positively or negatively influence a program’s implementation and the attainment of results [16].

Although the logic model traces its conceptual roots to program evaluation research [17–19], today its reach is far broader. It can guide program design, implementation, monitoring, operational research, and evaluation. During the last two decades, interest in the application of causal models has grown among academics, governmental agencies, non-governmental organizations, and practitioners of evaluation, primarily in industrialized countries [17]. The inclusion of this kind of causal thinking in the early stages of policy and program development is now making in-roads in non-industrialized countries [20].

### Overview

The purpose of this paper is to promote early and continuous causal thinking as decision makers design, implement, scale up, and evaluate CHW and other programs that are intended to positively affect the public’s health. The methods section describes how the generic CHW logic model was constructed, drawing explicitly on research in LMICs and the informed opinion of CHW experts with experience in these countries. The results section presents a graphic display of the model and detailed explanations of its component parts, in both narrative and tabular form. In the discussion section, the authors examine the value and unique contribution of the model and its potential as a tool to guide continuous learning about what works. They also present challenges of translating potential learning into tangible learning and describe some inherent limitations of the model. The paper concludes that, despite these challenges and limitations, the model offers the global health community greater clarity about how to think about, learn about, and ultimately support improved CHW performance.

## Methods: the process of model construction

The generic CHW logic model evolved over a two-year period (April 2011 to April 2013) through a multi-stage, inductive process. The different stages of the process were as follows.

### Initial planning/concept development

Model construction began during the initial planning of a structured review of the evidence on community and formal health system support for enhanced CHW performance, a US government-sponsored initiative led by the US Agency for International Development^a^. The organizers of the review adopted the following definition of a CHW: a health worker who receives standardized training outside the formal nursing or medical curricula to deliver a range of basic health, promotional, educational, and outreach services, and who has a defined role within the community system and larger health system. The first step in the planning process comprised formulating alternative definitions of CHW performance; identifying various factors with the potential to affect performance; and classifying common activities to support CHW performance by whether they were provided by health systems alone, by communities alone, or by both. To achieve these objectives in a short period of time, one of the authors [JN] performed a rapid qualitative content analysis of a small, purposive sample of recent (since 2010) key documents on CHWs [2, 3, 21–23]. The same author [JN] derived the definitions, factors, and support activities directly and inductively from this sample alone. This review was accompanied by a cursory exploration of gaps in the literature on efforts to improve CHW performance. The product of this formative work was an initial working conceptual framework.

### Evidence review

The organizers of the evidence review shared the working framework with approximately 50 CHW experts (representing academic institutions, bi-lateral and multi-lateral development agencies, and non-governmental organizations) who were invited to review the evidence on how best to support CHWs. The organizers subsequently assigned the experts to three evidence review teams and charged each with investigating a different question related to the three different sources of support for CHWs (community, health system, both). The preliminary conceptual framework was intended as a working template to guide the teams in their preparations for reviewing the literature and summarizing their findings. Each review team independently refined the template. The teams applied a variety of methods in doing so, including group discussion, literature review, analyses of CHW program case studies, and a modified theory of change exercise. Not all teams adopted all these methods, but most combined at least two of them. The teams incorporated visual displays of their respective frameworks into three draft evidence synthesis papers^b^.

### Expert consultation

A two-day expert consultation, which included more than one hundred professionals from around the world, provided each of the three review teams with an opportunity to share the findings of their independent reviews, including their frameworks. Although the organizers of the event did not explicitly ask the experts to comment on the three different frameworks, the experts’ oral and written feedback to the review teams about the review findings provided additional insights into the relevance and utility of the original working framework and each of the modified versions. A series of presentations from representatives of African and Asian countries that addressed the challenges of implementing and sustaining CHW programs at scale provided additional insights. At the conclusion of the consultation, the review teams revised their frameworks and draft synthesis papers, as necessary.

### Development of a synthesis model from the results of the evidence review, expert consultation, and supplementary analytical work

Once the review teams had submitted their final synthesis papers [24–26], two of the authors [JN and DF] completed a thorough content analysis of each with the intention of consolidating the diversity of conceptual thinking and findings toward the development of a single, cohesive, representative draft generic logic model. The three synthesis papers did not adequately address, however, how community systems can influence CHW performance. Consequently, the same two authors [JN and DF] consulted several key papers from the literature on building community capacity [27–29]. This cursory review produced a working outline on community systems, which all the authors examined for its relevance to CHW performance. The authors incorporated the information from this outline into a second draft of the generic logic model and continued to meet to refine the full model through discussion and further clustering and aggregating of information, which led to a series of additional iterations until the authors reached a consensus.

Ethical approval was not required as this study was based on reviewing published and unpublished literature and consulting with experts. A comprehensive description of all phases of the structured evidence review process, including its strengths and limitations, has been reported elsewhere [7].

## Results: the generic CHW logic model

**Figure 1:**
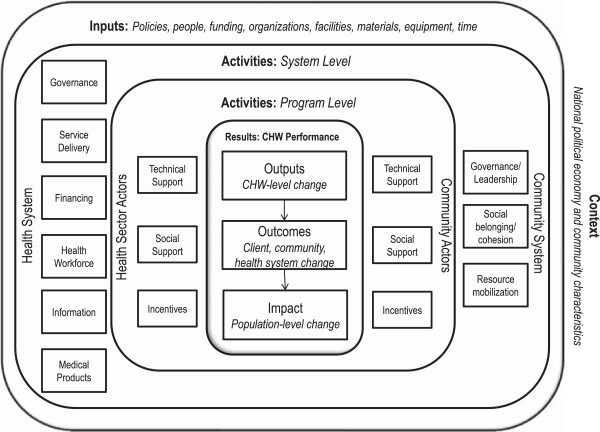
**Community Health Worker generic logic model.**

The generic CHW logic model (Figure 1) posits that optimal CHW performance (“Results”) is a function of high quality CHW programming (“Activities at the program level”), which is reinforced, sustained, and brought to scale by robust, high-performing health and community systems (“Activities at the system level”), both of which mobilize essential inputs and put in place processes needed to fully achieve the objectives of improved performance (“Inputs”). A range of contextual factors can also influence programming, system functioning, and performance. The component parts of the model are described briefly below, beginning with the intended results and working backward.

### CHW performance (“Results”)

The model depicts CHW performance in relation to the specific roles and responsibilities of CHWs in a given context in three ways: outputs, outcomes, and impact (Table 1). Outputs are proximate measures of performance that occur at the level of the individual CHW. Some are indirect measures, such as cognitive/psycho-motor (e.g., knowledge and skills acquisition) or affective (e.g., self-efficacy/self-esteem, confidence, or personal satisfaction) CHW-level changes, while others are direct behavioral measures that occur at the interface of CHWs and clients, such as absenteeism, the quantity and quality of service delivery, responsiveness to clients, and productivity. Attrition and advancement are measures of CHW developmental changes over time. Outcomes are intermediate measures, defined as CHW-attributable changes that occur among individual clients (e.g., health care-seeking behavior or health-promoting behavior in the home), as well as effects on communities and health systems (e.g., changes in social cohesion or cost savings to the health system, respectively). Impact refers to more distal measures, defined as CHW-attributable changes in health (e.g., morbidity and mortality) at the population level.

**Table 1:** **Measures and definitions of Community Health Worker performance in the generic logic model**

Results	Classification	Measures	Definition
**Outputs**	Indirect	Knowledge	Degree to which the CHW has the theoretical or practical understanding of the function and tasks assigned to him/her
		Competencies	Degree to which the CHW has the skills necessary to carry out the tasks assigned to him/her
		Motivation	An individual’s degree of willingness to exert and maintain effort on assigned tasks
		Morale	The mental and emotional condition (as of enthusiasm, confidence, etc.) of an individual CHW with regard to the function or tasks at hand
		Self-efficacy/esteem	A CHW’s confidence, belief in his/her ability to produce an expected, desired result
		Satisfaction	Degree to which CHWs derive personal satisfaction from serving the community, providing good quality services
	Direct	Absenteeism	Rate at which those CHWs who are supposed to be delivering services habitually fail to appear to carry out their tasks
		Service delivery	Quantity and quality of promotional, preventive, and curative services CHWs provide to community members
		Responsiveness	The degree to which an individual CHW responds to the needs of an individual client or group within a reasonable time period
		Productivity	A CHW’s total output per unit of total input
	Developmental	Attrition	The rate at which practicing CHWS resign, retire, or abandon their positions over time
		Advancement	The rate at which CHWs are advancing in their skills, competencies, formal responsibilities, and formal status within the community and the formal health system over time
**Outcomes**	CHW-attributable changes among individual clients	Access	Client’s physical and social access to essential services delivered by CHWs
		Knowledge of service availability	Client’s ability to identify the location of CHWs and the services they provide
		Health care-seeking behavior	Client in need of essential services and with access to CHWs is routinely seeking and using promotional, preventive and/or curative services CHWs offer
		Health-promoting behavior in the home	Client has adopted health-promoting behaviors in the home as a result of contact with CHWs
		Satisfaction	Client’s reported degree of satisfaction with the services rendered by CHWs
		Cost savings	Money not spent by client that he/she otherwise would have spent (on transportation and other items) in the absence of a CHW
		Health	Change in client’s state of illness, wellness, survival
	CHW-attributable changes in the community	Credibility	Degree to which the community considers CHWs to be making an important and valuable contribution to the health and well-being of the community
		Prestige	Status the community confers upon CHWs as a result of their selection and/or resulting from the quantity and quality of the services they deliver to community members
		Cost savings	Money not spent by a community that it otherwise would have been spent in the absence of a CHW to ensure its members secure health services
		Change in community functioning	Changes in a community’s structure, processes, and behaviors resulting from its interaction with a CHW
		Social cohesion	Change in the manner in which community members work towards achieving a goal or satisfy the emotional needs of its members resulting from its interaction with a CHW
		Community satisfaction	Community’s reported degree of satisfaction with the services rendered by CHWs
		Change in community health	Change in community’s state of illness, wellness, survival
	CHW-attributable changes in the health system	Credibility	Degree to which health system actors consider CHWs to be making an important and valuable contribution to the health and well-being of the community and the sound functioning of the health system
		Prestige	Status the health system confers upon CHWs as a result of their selection and/or resulting from the quantity and quality of the services they deliver
		Cost savings	Money not spent by the health system that it otherwise would have spent in the absence of a CHW to ensure the system was delivering high quality health services
		Change in health system functioning	Changes in health system structures, processes, and behaviors resulting from its interaction with a CHW
		Health system satisfaction	Health system actors’ reported degree of satisfaction with the services rendered by CHWs
**Impact**	CHW-attributable changes in health at the population level	Morbidity	Change in the prevalence of serious illness in the population served by CHWs
		Mortality	Change in the level of mortality in the population served by CHWs
		Fertility rate	The ratio of live births in a CHW-served area to the population of that area expressed per 1,000 population per year
		Equity	Degree to which access, coverage, or morbidity/mortality levels vary among different socio-economic or socially defined sub-groups in the population served by CHWs

### CHW programming (“Activities at the program level”)

Quality CHW programming comprises a wide range of support activities that explicitly target CHWs, seek to enhance their performance, and are undertaken by a range of actors in the health sector and the community (Table 2). These support activities are subsumed under three rubrics: technical support, social support, and incentives. Although these rubrics are common to both health sector actors (e.g., health workers, district health managers, etc.) and community actors (e.g., village health committees, local religious leaders, etc.) engaged in programming, some actors tend to carry out some of these activities more frequently than others, while other activities seem to be undertaken more equally by both. Of the three rubrics, technical support functions are those mostly commonly shared by health sector and community actors and thus are listed together in Table 2. For social support and incentives, however, the differences are more discernible; consequently, they are listed separately for health sector and community actors in Table 2.

**Table 2:** **The role of community and health sector actors in Community Health Worker (CHW) programming**

Technical support	Community and health sector actors	
1. Assist in CHW program design	Community and health sector actors participate in:	
	• Initial needs assessment to determine (1) demand for CHW services , as well as (2) views, perspectives, beliefs, and attitudes of all health sector actors affecting demand for CHW services	
	• Mapping of existing community-level services and identifying opportunities for aligning with CHW programs	
	• Developing a vision for CHWs that is shared by all stakeholders	
	• Identifying CHW selection criteria and methods of CHW recruitment	
	• Defining the service mix and package complexity*	
	• Developing a written program plan that includes the following elements:	
	(1) clear goals and objectives for the CHW program	
	(2) explicit roles, responsibilities, and expected competencies of CHWs (job description)	
	(3) a clear deployment plan to ensure adequate coverage	
	(4) specific activities CHWs are expected to implement	
	(5) a supervisory schedule and appropriate supervisory tools (e.g., performance checklists)	
	(6) an incentive scheme	
	(7) monitoring protocol for tracking CHW level of effort, including individual performance measures	
	(8) evaluation protocol for determining CHW program effectiveness, including program performance measures	
	(9) a budget	
	• Identifying existing and new referral mechanisms	
	• Identifying appropriate means to inform the community about the availability of CHW services	
	• Developing job aids and other tools that CHWs can use in providing services (such as health promotion activities)	
	• Developing new communication technologies that CHWs can use in providing and reporting on service delivery*	
	• Conducting a baseline assessment of CHW skills and capacity to inform CHW curriculum and training needs	
	• Developing or adapting a curriculum and materials to train CHWs	
	• Branding CHWs and their activities	
	• Briefing all stakeholders of the CHW programs (NGOs, private sector, local-level government) regarding their roles and responsibilities*	
	Sound design should take into consideration characteristics of CHWs (age, sex, literacy/numeracy, social and economic status, tenure as a CHW (years of service), degree of embeddedness in community and social networks, indigenous knowledge, mobility, residence, education, cultural belief system, ethnicity, religion, language, personal health behavior) and clients (age, sex, ethnicity, residence, education, religion, cultural belief system, socio-economic status, political affiliation).	
2. Assist in CHW program implementation and management	Community and health sector actors participate in:	
	• Conducting CHW Training of Trainers	
	• Training CHWs (pre-service and continuing, preventive and curative care skills, interpersonal communication and record-keeping skills)	
	• Orienting CHWs to local community context, as necessary	
	• Supervising CHWs	
	• Mentoring and coaching CHWs	
	• Providing continuous, constructive, contextually appropriate feedback	
	• Organizing and conducting demand-generation activities (via information sharing, education, communication, and advocacy)	
	• Helping to organize/coordinate/manage service delivery events (household and community: health fairs, educational sessions)	
	• Mobilizing local material support and resources for CHWs for the short- and long-term**	
	• Arranging for transport of clients in emergency situations**	
	• Ensuring positive client-CHW interactions	
	• Ensuring functioning supply system for timely and sustainable availability of essential drugs, commodities, supplies, equipment, materials (including for record-keeping), tools, and technologies*	
	• Ensuring logistics support	
	• Organizing/coordinating service delivery events (household and community: health fairs, educational sessions)*	
	• Ensuring proper financial management of CHW program funds	
3. Assist in program monitoring and evaluation (M&E)	Community and Health Sector actors participate in:	
	• Developing monitoring and evaluation protocols*	
	• Monitoring CHW level of effort through continuous collection of information (via meetings, household visits, etc.) about quality of CHW service delivery and the community’s access to, acceptability of, and satisfaction with services	
	• Archiving information about CHW service delivery within local HMIS*	
	• Evaluating CHW’s individual performance and overall program effectiveness	
	• Providing continuous and appropriate feedback to CHWs, community, and health sector, including sharing information about best practices and lessons learnt from program implementation	
	• Continuously adapting the program, as necessary based on M&E results	
**Social Support**	**Community Actors**	**Health Sector Actors**
Develop partnerships, strengthen linkages, and enhance networks	Assist CHWs in developing mutually reinforcing partnerships with formal structures and actors outside the community to support CHW program design, implementation, and M&E activities:	Assist CHWs in developing mutually reinforcing partnerships with formal structures and actors outside the health system to support CHW program design, implementation, and M&E activities:
	• Health Sector actors	• Community system structures and actors
	• Government at all administrative levels	• Structures and actors in other government sectors
	• Media and journalists	• Media and journalists
		• NGOs, community-based groups
	Orient CHWs and assist them in developing productive linkages with existing and new structures and actors (both health and non-health) within the community to support CHW program design, implementation, and M&E activities:	Orient CHWs and assist them developing productive linkages with actors within the health system in helping to support CHW program design, implementation, and M&E activities:
	• Community leaders (who can raise broader community awareness and acceptance of and support for CHWs and their services)	• Align roles and responsibilities of CHWs with those of other health system health care providers
	• Village health and development committees, advisory groups, coordination and oversight bodies	• Integrate CHWs into formal health system by incorporating them in sub-systems for health workforce development (training and supervision), service delivery (ensuring functioning referral system), and logistics management
	• Women’s groups	• Integrate CHW into health care service delivery teams
	• Religious groups	• Promote and market CHW services within the formal health system to ensure health workforce buy-in for CHWs
	• Faith-based organizations	• Provide continuous support to CHWs and manage potential conflict between CHWs and health professionals
	• Community-based organizations	• Recruit health professionals to staff health committees, oversight bodies, and advisory groups to provide support and feedback to CHWs and communities
	• Non-governmental organizations	
	• Traditional structures and indigenous practitioners	
	• Local civic and social clubs	
	• Savings groups/loan associations	
	• Schools	
	Assist CHWs in strengthening their professional and personal networks:	Assist CHWs in strengthening their professional and personal networks:
	• Facilitate CHW peer exchange and/or membership and participation in CHW associations to improve peer support	• Facilitate CHW membership and participation in CHW associations to improve peer support
	• Recognize and applaud family, kinship group, and other community member support to CHWs	• Recognize and applaud family, kinship group, and other community member support to CHWs
	• Publicly promote, market CHW role and services	
**Incentives**	**Community Actors**	**Health Sector Actors**
1. Non-financial	• Community actors demand CHW services and expresses satisfaction with these services	• Health Sector actors demonstrate publicly their appreciation of and respect for CHWs (via health system awards, annual days of honor and recognition, etc.)
	• Community actors express its appreciation for and praises CHW activities and achievements	• Health Sector actors ensure public visibility of CHWs (e.g., by posting photos of CHWs and other branding activities)
	• Community actors accept, endorse, and trust CHWs	• Health Sector actors provide learning and development and career advancement possibilities to increase CHW social status
	• CHW is elected by community actors to represent it on local councils and other decision-making bodies of influence	• Acceptance by formal health system health care providers (i.e., legitimization of the role and value of CHWs)
	• Community provides CHWs with opportunities for self-improvement, increased social interaction and mobility, meaningful income, or further training	
	• Community provides constructive feedback to CHWs, community members, community groups, and government actors about CHW performance	
	• Community elevates CHWs’ status within the community	
2. In-kind	• Special privileges: exemption from other community duties, access to free social services, etc.	• Special privileges: health system funds health and social activities of communities served by CHWs
	• Goods: animals, food, gifts, etc.	• Goods: branded umbrellas, bicycles, motorcycles, uniforms, badges, mobile phones, stationary, etc.
	• Services: farm labor, finance with local resources health activities led by CHWs, etc.	• Services: provide CHWs with free or preferential access to health care; psychological support for CHWs and family members
	• Equipment: branded umbrellas, bicycles, badges, uniforms, stationary, mobile phones, etc.	
3. Financial	• Cash compensation for services rendered (e.g., fee for service)	• Permit CHWs to draw supplementary income/modest profit from sale of medicines, commodities, and other health-related products
	• Direct and regular salary payment from community health structures	• Provide cash compensation for services rendered (e.g., fee for service)
	• Direct and regular stipend from community health structures	• Provide some portion of CHWs’ direct and regular salary payment from formal health system structures
	• Allowance/benefit for transport or training	• Provide direct and regular stipend from health system structures
	• Performance-based financial reward (where deemed appropriate)	• Provide allowance/benefit for transport or training
	• Access to micro-credit funds	• Provide performance-based financial reward (where ]deemed appropriate)

*health sector actors only; **community actors only.

*Technical support* includes efforts by health sector and community actors to design good CHW programs, ensure sound program implementation and management, monitor adequacy of effort, and evaluate effectiveness. Sound programming also takes into consideration the characteristics of both CHWs (e.g., age, sex, ethnicity, education, experience) and their clients (e.g., socio-economic status, cultural belief system, education, age, sex).

*Social support* includes various activities that health sector and community actors undertake with non-health and health sector representatives to enhance CHW programming. For example, these activities may include health sector and community actors fostering new partnerships with non-health sector structures and representatives such as political, administrative, and government officials, journalists, non-governmental organizations (NGOs), and social action community-based groups. Other activities are intended to strengthen the linkages with various groups that have a history of working on health promotion and disease prevention activities, such as faith-based organizations, NGOs, social and civic clubs, savings groups and loan associations, women’s groups, schools, health care delivery teams, district health offices, and health oversight bodies. Continuously promoting and developing the professional and personal networks of CHWs, such as CHW associations and peer groups, is another area of targeted social support. As Table 2 demonstrates, health sector and community actors often perform these social support activities in different ways.

*Incentives* encompass non-financial (e.g., gestures reflecting community appreciation of and trust in CHWs), in-kind (e.g., specific privileges, goods, and services), and financial inducements (e.g., fees for service, salary, stipend, or allowances/benefits) that are commonly used to motivate CHWs to enhance and sustain their performance. Although there is some overlap between incentives, particularly the non-financial ones, and social support, they are described separately to adequately capture the many dimensions and nuances of inducements as an important source of support for CHW performance. Again, community and health sector actors vary in their performance of these functions.

The model assumes that all these program-level activities, when adequately implemented in terms of both intensity and quality of effort, will contribute to improved CHW performance.

### Systems support (“Activities at the system level”)

In contrast to the targeted nature of CHW programming, performance-enhancing activities at the level of health and community systems have multiple and synergistic direct and indirect effects across numerous health sector programs and providers (i.e., not limited to CHWs). Consequently, in contrast to the common rubrics of CHW program support activities, there is greater variation in how health system and community system support activities manifest themselves and influence CHW programming and ultimately CHW performance (Table 3).

**Table 3:** **The role of community and health systems in reinforcing Community Health Worker programming**

Community system elements	
**Leadership/Governance**	
1. Vision	Leadership articulates a clear vision for achieving health and development outcomes for the community
2. Service and resource availability	Leadership identifies all curative and promotional health and social services available to the community and their social accessibility to community members
3. Equity	Leadership ensures vulnerable and disenfranchised groups have equitable access to essential health and social services
4. Collective action	Leadership ensures collective processes and actions that can promote the community’s health and development
	• Mobilizes community assets to engage in key policy, legal, and governance activities (such as campaigns, solidarity movements, and other advocacy actions)
	• Ensures participatory decision-making by actively engaging community members in identifying problems and concerns, implementing their plans to solve these problems, and taking responsibility for their actions
	• Facilitates consensus-building and collaboration that fosters trust, respect, negotiation, openness, conflict resolution, creativity, and responsibility among members
	• Identifies areas in which community groups and members need to make changes in the way they work together and provides guidance and support in making these changes
	• Respects and values the viewpoints of community members and cultivates community input and action
	• Ensures transparency and accountability through meetings and other means of communication with stakeholders and community members
	• Manages power relationships within and beyond the community to promote community development and well-being
	• Fosters ownership over team decisions by suggesting new ideas, expressing opinions, and pointing out ways to overcome obstacles
5. Knowledge management	Leadership acknowledges, documents, and disseminates individual and community achievements and challenges encountered in improving the community’s health
6. Mentoring	Leadership fosters the development and emergence of new leaders and other assets
7. Sustainability	Leadership ensures any successes in improving the community’s health can be sustained beyond short-term projects:
	• Sustains a program’s focus of activity and gains funding and resource commitments
	• Encourages the development of mutually reinforcing partnerships with formal health and development structures and actors beyond the community
	• Supports strengthening productive linkages with groups within the community
	• Encourages and cultivates self-help activities
**Social belonging/cohesion**	
1. Trust/belonging	• Community members exhibit trust among group members and feel part of the community
	• Community members have positive perceptions of their communities, value their diversity, celebrate together, and have a sense of control and ownership in relation to planning and implementing local programs and activities to improve their health and well-being
2. Historical perspective	Community members understand the community’s history
3. Compassion	Community members show a sense of compassion for others in the community
4. Identify	Community members have a shared identity and are willing to take action based on that identity
5. Commitment	Community members have a commitment to achieving outcomes and positive change and a shared responsibility for improving the community
**Resource mobilization**	
1. Identification	The community routinely identifies external and internal resources (funding, people, organizations, facilities, material, time) to help achieve its health goals
2. Procurement	The community routinely accesses external and internal resources (funding, people, organizations, facilities, material, time) to help achieve its health goals for the community
3. Use	The community uses resources (funding, people, organizations, facilities, material, time) in new, creative, and effective ways to achieve its health goals
4. Allocation	The community makes informed decisions about fair distribution of resources and resolves conflicts regarding distribution, including distribution of common resources
**Health system elements**	
1. Governance	• Formulates and aligns all health sector strategies and technical policies
	• Identifies roles of public, private, and voluntary health system actors and of civil society at central and decentralized levels of the health system
	• Provides robust oversight and regulation of health markets and all health activities in the public and private sector
	• Holds all health system actors in the public and private sectors accountable for activities and results
	• Provides incentives that reward good performance and sanctions poor performance to all health system actors in the public and private sector
	• Ensures collaboration and coordination across sectors in government and with actors outside government
	• Ensures generation, analysis, and use of intelligence on health sector performance trends
2. Financing	• Raises adequate funds for the health sector
	• Allocates these funds in accordance with population needs and in ways that ensure people can use needed services
	• Pools funds when possible to ensure people are protected from financial catastrophe or impoverishment associated with having to pay for services
	• Purchases packages of high-quality, high-impact services
	• Promotes transparency and accountability in financing systems
	• Ensures generation, analysis, and use of intelligence on the performance of the health financing system
3. Health workforce	• Develops national workforce policies and investment plans
	• Defines the roles, responsibilities, and performance expectations (as stated in service agreements or contracts, for example) of all health workers
	• Ensures appropriate recruitment and development of the workforce (skill mix/cadre development)
	• Ensures appropriate deployment and distribution of health workers relative to fixed facilities and burden of disease
	• Uses strategic information to monitor the availability, distribution, and performance of health workers
	• Establishes regulatory mechanisms to maintain the quality of education/training and practice
	• Engages with multiple stakeholders and sectors for human resources for health (HRH) planning and workforce development
	• Develops retention schemes that take into consideration local and international labor markets
	• Designs training programs and other capacity development activities that facilitate integration across service delivery and disease control programs
4. Service delivery	• Organizes and regulates the health care delivery system in a way that ensures delivery of effective, safe, quality personal and non-personal health interventions to those who need them, when and where needed, with minimum waste of resources
	• Develops an organized provider network to ensure close-to-client care
	• Adapts, adopts standard practice guidelines for the delivery of essential services in line with the HRH plan
	• Delivers package of integrated services based on population health needs
	• Generates demand for services through an understanding of the user’s perspective, raising public knowledge, and reducing barriers to use (cultural, social, financial, gender, etc.)
	• Ensures proper management of service delivery at all levels to maximize service coverage, quality, safety, and minimize waste, including supervision, performance incentives, and a functioning referral system
	• Oversees infrastructure and logistics (i.e., buildings, utilities, waste management, transport, communication)
5. Medical products, vaccines, and technologies	• Ensures equitable access to essential medical products, vaccines, technologies, equipment, and supplies of assured quality, safety, efficacy, and cost-effectiveness by:
	○ Developing national policies, standards, guidelines, and regulations in accordance with local laws
	○ Setting and negotiating prices, using information on prices and international trade agreements
	○ Ensuring reliable manufacturing practices and quality assessment of products
	○ Developing procurement, supply, storage, and distribution systems
	• Promotes rational use of essential medicines (drugs, vaccines), commodities, technologies, equipment, and supplies
6. Information	• Ensures the collection (via population-based, facility-based, and special surveys), analysis, dissemination, and use of timely and high quality information on:
	○ Health status
	○ Financial risk protection
	○ Health service use
	○ Client satisfaction with services
	○ Health behavior
	○ Health system performance
	○ Events that threaten public health security
	• Ensures long-term capacity to archive and manage information, as well as promote its availability in the public domain and application

Robust-performing health systems can reinforce CHW programming, sustain results, and take efforts and effectiveness to scale through sound governance of the sector; timely and adequate sector financing; well-organized service delivery; ensuring a capable and well-deployed health workforce; the systematic collection, analysis, and use of information; and ensuring access to a broad range of medical products and commodities [30]. For their part, communities contribute to quality CHW programming through sound governance of community resources; ensuring social belonging and cohesion; and resource mobilization [27]. As noted previously, the model also assumes adequacy of effort at the system level: activities are timely, comprehensive, and of sufficient quantity and quality.

### Inputs

For each of the many activities at the program and system levels in the model there is an input implication. For the sake of parsimony, the authors have included only the main rubrics in the model—written policies and programs, people, funding, organizations and facilities, material and equipment, and time (Figure 1). Again, the model assumes adequate levels and timely availability of these resources (quantity and quality) at program and system levels to achieve improvements in CHW performance.

### Contextual factors

Many contextual factors can influence CHW programming, systems functioning, and CHW performance. Some of these factors are associated with the larger political economy of a country, whereas others relate to the characteristics of communities that CHWs serve. Some examples of political economy include the structure, rules, dynamics, and balance of power in society; the role and influence of interest groups in national decision making; the role of foreign aid in development; the degree of tolerance of corruption; the extent of transparency in governance; frequency of political elections, violence, and coercion; and the level of ethnic fragmentation in society. Examples of community characteristics are cultural values, geography (urban/rural), economic status (poverty level), social status (isolation, discrimination, gender norms), stability (nomadic, transitional, permanent), health belief system (preference for traditional medicines), and history of and experience with volunteerism.

## Discussion

### Value of the model

This multi-level model is a unique contribution to current thinking about CHWs and their performance for several reasons. It places performance and how to improve it at the center of the discussion about CHW programming, which is increasingly expected to make important contributions to the achievement of results-oriented Millennium Development Goals- and Universal Health Coverage-related initiatives at global and country levels. Additionally, it recognizes that the different contributions of both health sector and community actors to high quality CHW programming are necessary but not sufficient to attain and sustain CHW performance at scale. CHWs find themselves at the intersection of two overlapping and dynamic systems, both of which have a critical and mutually supportive role to play in enabling and reinforcing CHW programming. If today’s CHW programs are to overcome the weaknesses of CHW programs of the past [31], strong health and community systems will be needed to ensure the sustainability of health gains achieved through discrete targeted programs.

Furthermore, the model is integrative in that it draws on a broad range of the available literature, both published and unpublished, first-hand stakeholder experience, and the informed opinions and perspectives of experts. Finally, the authors adopted an innovative, pragmatic approach to logic model construction that combined certain elements of traditional approaches (single analyst and collaborative group process) [17] that were adapted to the unique circumstances under which this work was to be carried out. The approach was largely opportunistic, iterative, drew on a range of sources, and was embedded in a larger evidence synthesis exercise carried out in a compressed timeframe. The process began with convergent thinking toward the development of a preliminary working conceptual framework, subsequently evolved into divergent thinking as reflected in review teams’ multiple variations of the original framework, and ultimately returned to convergent thinking in pursuit of a single, comprehensive, framework. Although this convergence-divergence-convergence cycle was a time-intensive process, the benefit was a more robust identification and rendering of the many determinants of CHW performance.

### Utility of the model

The generic CHW logic model not only represents a novel approach for how to think about improving CHW performance, but also offers a framework that decision-makers, including policymakers, planners, researchers, and communities, can use to learn about what does and does not work in practice. The generic CHW logic model can serve multiple, pragmatic purposes. First, it can be an aid to planning. Decision makers can consult and adapt the model when developing or modifying local CHW policies and programs. The value of a generic, comprehensive model is that it can draw decision-makers attention to certain elements of sound design that are sometimes overlooked. For example, this model highlights the important role of community systems, which is inadequately addressed by the published literature, yet repeatedly mentioned in the gray literature and by experts as a significant determinant of good CHW performance. Hopefully, the model will stimulate planners to take into account the contribution of community systems in optimizing programming and sustaining CHW performance.

Second, use of the model can contribute to consensus-building. The use of local program theories, which allow decision makers to describe, in explicit terms, how they expect their CHW programs to work, can facilitate communication among program developers, researchers, policy makers, community representatives, and funders. Improved communication can help foster a shared understanding of the depth and breadth of what is needed to improve and sustain CHW performance [16, 32, 33], and promote a common understanding among country staff and community representatives of their shared mission, a critical first step in developing a mutual accountability framework. This shared understanding can inform the current dialogue about the need to address fragmented stewardship of CHWs at both country and global levels [34, 35].

Third, as decision-makers adapt the model to local conditions and available resources, they can use it as a guide for improving program implementation. The assumed linkages and relationships represented in country-specific causal models can generate a series of questions that can be explored through routine monitoring, systematic documentation of implementation (preferably prospective), and other forms of systematic inquiry around implementation such as operational or action research. Observation and documentation of the actual intensity and adequacy of intended implementation – an explicit assumption of the generic model – as well as short- and medium-term intended and unintended effects of implementation, can improve management and contribute to continuous learning about why a CHW program does or does not work. The answers to these questions may result not only in improvements to programming, but also in modifications to the assumptions explicit and implicit in local and generic models.

Fourth, a local theory of the program adapted from the generic logic model, if used to guide summative evaluation, can contribute to collective learning about program effectiveness [18, 19]. Summative evaluation responds to senior policy makers’, funders’, and community leaders’ end-game concerns about whether the program worked, what it was about the program that led to success, and whether they should invest further to extend its reach or take it to scale. Documentation of the extent to which a local program theory has been implemented as intended, when incorporated into evaluation designs, can provide some support for attributing the observed outcomes to the program even in the absence of a counterfactual [17] – a common limitation in the literature on support strategies for CHW performance.

### Future challenges

How can this potential for learning be translated into tangible learning? There are several practical challenges in moving forward. The most pressing is to find an opportunity to test the assumptions of the generic CHW logic model under real-life conditions. To that end, the authors have commissioned a retrospective study, using the logic model as a framework for analysis, of the factors associated with the success of a growth monitoring and promotion program implemented by female village volunteers in Honduras. The authors also have commissioned field research in a sub-Saharan African country to determine the content and face validity of the model to ascertain its relevance as a framework for identifying strengths and weaknesses of current efforts to improve CHW performance, as well as for identifying new opportunities for doing so. These activities should provide much-needed insights on the validity of the model and its generalizability to different settings.

A second challenge is determining the feasibility of local adaptations of the generic model in routine policy making and programming. Although the model in Figure 1 appears simple and straightforward, CHW policy makers and program managers may perceive the underlying number and variety of elements and relationships as depicted in the tables to be intimidating. An inter-country, continuous learning exchange, whereby CHW stakeholders periodically share experiences about selected causal links in their respective models at different points in time can provide ideas and support for innovation that may partially address this challenge. One benefit of this kind of south-to-south knowledge management initiative would be a more systematic and user-friendly gray literature derived from documentation of program implementation framed by explicit logic models, which could significantly contribute to the global community’s collective understanding of what works under different conditions. Furthermore, documentation of the costs and benefits of holding these adapted models accountable for real-world results can inform global efforts to ensure adequate stewardship of donor support to national efforts and in building national capacity.

A third challenge is ensuring the generic model is adapted to local conditions, and that local adaptations and the generic model are updated in response to changing realities. The factors influencing CHW performance are complex; any generic logic model will be an imperfect and oversimplified reflection of reality and at best a snapshot of that reality at a single point in time. For example, a generic model may not offer the precision needed to differentiate the performance determinants of volunteer CHWs working on HIV and AIDS from those of paid CHWs working on improving access to curative care. No single, generic CHW logic model will be equally relevant to all countries: the genesis, purpose, evolution, and complexity of CHW programs around the world can differ substantially from one country to another. Furthermore, the inputs and activities required to sustain a fully functioning program will vary in type, mix, intensity, and sequence across countries. Therefore, generic models must be adapted to local conditions and evolve as circumstances change. For instance, the use of mobile technology may alter our current understanding of what is needed to adequately support CHWs. Continuously building better generic and adapted models based on feedback from multiple information sources is both a challenge and an opportunity for understanding better what works in practice in different settings.

### Limitations

The generic CHW logic model has several limitations. First, all the elements in the model appear equally weighted. At this time, there is no strong evidence base to support weighting, yet weighting will likely vary from context to context. The Honduras study should shed some light on the relative contributions of different support strategies to improved performance in this particular setting and with this particular cadre of CHWs. Further research of this kind, however, is urgently needed.

Second, the model should not be interpreted as normative guidance for how to improve CHW performance. It is a working theory in the absence of strong scientific evidence on the definitive causal pathway to improved performance. This does not mean that the different activities reviewed and included in the model do not work or do not merit support. It means that continuing implementation of promising activities expected to influence performance should be accompanied by prospective monitoring and documentation of the adequacy of effort expended, the influence of many factors, and any intended and unintended effects. More rigorous research should be conducted when and where possible.

Third, although use of the model can inform programming, by itself it is not an adequate program planning tool. A “logical framework” or “log frame” is a natural extension of the logic model: it magnifies the logic model by integrating indicators and targets and the means for measuring progress [36, 37]. The additive components of a log frame make it an indispensable complementary management tool for program planning, monitoring, and evaluation of specific country CHW programs. Likewise, the logic model is primarily descriptive, not explanatory. For the expected linkages it does propose, the logic model does not unpack the underlying assumptions in the causal results chain. The “theory of change” approach is better suited to this kind of explanatory work [38]. It provides a thorough analysis of ‘why’ program activities are expected to produce intended results and creates a more in-depth understanding of ‘how’ change can occur [39].

Finally, the logic model concept is based upon a general systems theory approach to management [40], which is consistent with the literature and expert opinion on CHW performance that suggest that support activities across and within health and community systems are not linear, but rather interdependent, and may interact to achieve the intended results [26]. The two-dimensional graphic representation of the CHW logic model (Figure 1) is not capable of capturing this dynamism adequately [38].

## Conclusions

A robust evidence base for developing a definitive causal pathway to improved CHW performance does not yet exist. Although such a pathway may remain aspirational, the importance of CHW performance in today’s results-oriented environment should spur further development of such pathways. This generic CHW logic model partially addresses this knowledge gap by proposing a theoretical causal pathway to enhanced performance.

The model posits that robust, highly functioning health and community systems enable and reinforce CHW programming, which offers the prospects of sustaining CHW performance at scale beyond the life of discrete CHW-targeted programs and projects. By examining both formal health and community system support for CHWs in an integrated manner, the model highlights the multi-level and multi-dimensional challenges and complexity of enhancing CHW performance. The model is a novel contribution to current thinking about CHWs. It places CHW performance at the center of the discussion about CHW programming. It highlights the strengths and limits of targeted programming, and is comprehensive, reflecting the current state of both scientific and tacit knowledge about support for improving CHW performance. The authors adopted an innovative, opportunistic approach to model construction, combining specific elements of traditional approaches and embedding them in a larger evidence synthesis exercise.

The model also offers a comprehensive framework to stimulate continuous learning about what works. It is a cause for concern that CHW programs continue to proliferate, go to scale, and grow in the absence of routine monitoring information and strong research evidence on what support activities, or combination of activities, work best. It is critical to pay more attention to answering the question of how best to enhance CHW performance at scale and to guide investment decisions of governments, communities, and donors alike in this time of transition in development assistance for health. The CHW generic logic model can make an important contribution to this learning agenda despite several challenges in translating the potential for learning into actual learning, and some inherent limitations in the model. It suggests a way forward, yet leaves ample space for continuous modification, creativity, and innovation.

### Endnotes

^a^Although it can be argued that communities are important actors in producing health, and therefore could be considered as part of the health system, the organizers of the evidence review chose to separate the community’s contribution to improving CHW performance precisely to highlight the important and often overlooked role communities play in this process.

^b^In all, the three teams reviewed 147 documents and summarized the evidence in terms of what was known, what remained to be investigated, and what recommendations for action could be drawn. A complete bibliography is available from the authors upon request.

## Authors’ information

JN is the Health Systems Research Advisor in the Office of Health Systems, Global Health Bureau, USAID, Washington, DC, on assignment from the U.S. Centers for Disease Control and Prevention (CDC), Atlanta, Georgia. DF is a Health Systems Strengthening Advisor in the Office of HIV/AIDS, United States Agency for International Development (USAID), Washington, DC. TW is a Senior Improvement Advisor for Health Workforce Development for the USAID Applying Sciences to Strengthen and Improve Systems Project (ASSIST) at University Research Co., LLC (URC), in Bethesda, Maryland. LMF is Vice-President for Technical Assistance and Evaluation for EnCompass LLC, in Rockville, Maryland. MN is Vice-President, Global Health and WASH, World Vision International, in Washington, DC.

## Abbreviations

CHW: Community Health Worker
LMICs: Low- and middle-income countries.
